# “Reality rarely looks like the guidelines”: a qualitative study of the challenges hospital-based physicians encounter in war wound management

**DOI:** 10.1186/s13049-018-0517-y

**Published:** 2018-06-27

**Authors:** Andreas Älgå, Karin Karlow Herzog, Murad Alrawashdeh, Sidney Wong, Hamidreza Khankeh, Cecilia Stålsby Lundborg

**Affiliations:** 1 Department of Clinical Science and Education, Södersjukhuset, Karolinska Institutet, Stockholm, Sweden; 2 Department of Public Health Sciences, Karolinska Institutet, Stockholm, Sweden; 3 Médecins Sans Frontières, Ar Ramtha, Jordan; 4 Médecins Sans Frontières, Operational Centre Amsterdam, Amsterdam, The Netherlands; 5 Health in Emergency and Disaster Research Center, University of Social Welfare and Rehabilitation Sciences, Tehran, Iran

**Keywords:** War wounds, Perceptions, Antibiotic resistance, Healthcare-associated infections, Qualitative study

## Abstract

**Background:**

Globally, armed conflict is a major contributor to mortality and morbidity. The treatment of war-associated injuries is largely experience-based. Evidence is weak due to difficulty in conducting medical research in war settings. A qualitative method could provide insight into the specific challenges associated with providing health care to injured civilians. The aim of this study was to explore the challenges hospital-based physicians encounter in war wound management, focusing on surgical intervention and antibiotic use.

**Methods:**

Semi-structured, face-to-face interviews were conducted with physicians at a Jordanian hospital supported by Médecins Sans Frontières. The interviews were recorded, transcribed verbatim and analysed using content analysis with an inductive and deductive approach.

**Results:**

We found that challenges in war wound management primarily relate to protocol adherence. Protocols for the management of acute war wounds were adhered to on areas that could be considered commonly agreed principles of war wound surgery, such as the use of wound debridement and the evaluation of the systemic condition of the patient before initiating antibiotic treatment. We identified limitations imposed on the physicians that complicate or even hinder protocol adherence. Additionally, we identified factors associated with conscious deviations from the protocols.

**Conclusions:**

We conclude that adherence to established protocols around the management of acute war wounds is difficult. We present aspects that may be considered when establishing clinical projects in similar contexts. The knowledge gained by this study could provide insights for the development of protocols or guidelines for wound management and antibiotic use in an unstable setting, such as a hospital in close proximity to armed conflict. We suggest the use of a grounded theory approach to further study the discrepancy between guideline recommendations and actual practice.

## Background

Armed conflict has a major effect on global public health and result in substantial mortality and morbidity [1]. War-associated wounds are often grossly contaminated, leading to an increased risk of infection [2]. Surgical intervention and antibiotic prophylaxis limits infectious complications. Still, infection remains a major threat to life and recovery of function in war wounded [3, 4]. War wound surgery is performed according to commonly agreed principles. Due to the limited available research, these principles are largely experience-based. The surgical treatment heavily relies on clearing the wound of devitalised tissue and contaminating material, so called debridement [5]. Antibiotics are used both as perioperative prophylaxis and as part of the treatment of wound infections [6]. However, the efficacy of antibiotics is threatened by globally escalating antibiotic resistance [7], driven by the widespread overuse and misuse of antibiotics [8]. War wounds are often infected by antibiotic resistant bacteria, further complicating the management [9, 10].

The Syrian civil war has claimed over 280,000 lives to date [11]. Over five million people have fled the country, many to neighbouring countries [12]. Currently, the lower-middle-income country Jordan [13] hosts 655,000 Syrian refugees [12]. Many refugees have sustained war-associated injuries, predominantly due to blasts and gunshots. The management of these injuries is filled with challenges, related mainly to high numbers of severely injured patients, resource scarcity, and a lack of evidence-based treatment guidelines. Yet, limited research exists on the experiences of the treating physicians and what are perceived as the main challenges. In this study, the relatively stable Jordanian setting, in combination with the proximity to the Syrian armed conflict, provided us with a unique opportunity to study challenges in the complex management of acute war wounds, without the practical and ethical difficulties usually associated with research in such close proximity to armed conflict. To understand the way physicians perceive the challenges in this context we concluded that a qualitative study would be most suitable. Qualitative methods are used to generate knowledge from a context-specific perspective by exploring people’s experiences and perceptions [14]. The method of interviews and subsequent content analysis has been shown to be effective in the investigation of an un-explored area by letting participants freely present their views and elaborate on a topic [15]. The aim of this study was to explore the challenges hospital-based physicians encounter in the management of war wounded civilians, focusing on surgical intervention and antibiotic use.

## Methods

### Study setting

Since 2013, Médecins Sans Frontières/Doctors Without Borders (MSF) has been running an emergency trauma project at the Ministry of Health hospital in Ar Ramtha, Jordan, near the Syrian border [16]. Nurses and non-specialised physicians working at the emergency department and the wards are predominately from Jordan. The specialist physicians, general surgeons, orthopaedic surgeons, and anaesthesiologists, are roughly 75% from Jordan and 25% MSF expatriates, who originate from countries around the world. Management positions are primarily held by MSF expatriates.

Patients are civilians from the Syrian armed conflict, who receive treatment according to guidelines based on the International Committee of the Red Cross (ICRC) war surgery protocol [5]. As prophylactic treatment, narrow-spectrum antibiotic agents are used as an adjunct to thorough wound debridement. Acute war wounds are left open following the initial surgery. After 3–5 days, the wound is assessed and treated by delayed primary closure if possible. Wound infection rate among surgically treated patients at the project has been found to be 11%, with three of four patients infected by multidrug-resistant bacteria [10]. The physicians’ views and unique experiences on the challenges around wound healing and antibiotic use have not been systematically explored.

### Participants

Practicing physicians directly involved in war wound management at the MSF project in Ar Ramtha hospital were selected. The selection was made using purposeful sampling directed towards achieving maximum diversity in wound management experiences, aiming at heterogeneity in age, sex, country of origin, medical specialty, years of work experience, and further education on antibiotic prescription and resistance. Participant demographic data is given in Table 1.

**Table 1:** Characteristics of the ten participants

Characteristic		
Age, mean (range)	37	(27–63)
Male, n (%)	6	(60)
Origin, Jordan (non-Jordan)	6	(4)
Medical speciality, general or orthopaedic surgeon (not specialised)	5	(5)
Years of working experience, mean (range)	11	(2–35)
Further education on antibiotic resistance, yes (no)	3	(7)
Further education on antibiotic prescription, yes (no)	0	(10)

### Data collection

The research group constructed an interview guide with questions and probing areas (Appendix 1). Semi-structured, individual, face-to-face interviews were conducted and tape-recorded during 2015 in Ar Ramtha hospital. All interviews were performed by the first author (AÄ), who was able to observe each interviewees’ body language and gestures. AÄ is a Swedish general surgery resident that has worked primarily in Sweden, but also has MSF field experience abroad. AÄ was not known to the participants of this research prior to undertaking the study. The interview guide was discussed within the research group and adjusted in-between interviews. The participants were included until saturation was reached and further data collection did not contribute new information to the main topics.

Eleven physicians were invited to participate and all consented to the study. In one interview the recorder malfunctioned and consequently the interview was excluded. One interview had to be terminated in advance because the participant was needed in the hospital. This interview is therefore short but was included in the analysis process. In total, ten interviews were analysed. The interview duration ranged from 13 to 50 min with a mean duration of 32 min.

### Analysis

A qualitative approach using content analysis was utilised to explore the experiences and perceptions of treating physicians in their work with war-associated wounds. The interviews were transcribed verbatim. The transcribed interviews were read several times in their entirety to grasp the overall content of the interview and to correct mishearing or misinterpretations.

Analysis of both manifest and latent content with an inductive and deductive approach was used, as described by Graneheim and Lundman [17]. The manifest content is the concrete meaning of the text, whereas latent content is the interpreted underlying meaning. The inductive approach consists of condensation and abstraction, step by step, in order to derive the broader meaning from the interview material. The deductive approach uses a pre-existing perception, theory or guideline for continuous comparison when the interview material is condensed and abstracted. Protocols for wound care, antibiotic treatment, and hygiene from the MSF project in Ar Ramtha were used for the deductive analysis. For the content analysis, meaning units, defined as words, sentences or paragraphs related by their context or meaning, were first identified. The meaning units were then condensed if needed. Subsequently, codes, sub-categories and categories were developed by both inductive and deductive approaches using manifest content analysis. The main theme was derived through latent content analysis. Continuous guidance by experienced qualitative researchers was ensured throughout the coding and analysis process. Analysis was conducted primarily using tables in Microsoft Word^®^ 2011 (Microsoft, Redmond, Washington, USA). An extracted example of the coding and analysis process is shown in Table 2.

**Table 2:** An example of the process of condensation and abstraction, from meaning unit to category

Meaning unit	Condensed meaning unit	Code	Sub-category	Category
“Clinically, we would just check the wound.” 5^a^	Local infection, assessed by clinical wound investigation	Local symptoms of infection	Wound assessment and treatment of infection	Adherence to protocols and commonly agreed praxis
“So, each time we do change of dressing we will notice these things because we will write down if it’s clean, if it’s dirty and it will affect the plan for how long we are going to doing the… how long we are doing the change of dressing.” 5^a^	Wound assessed for infection in every dressing change, it’s written down and affects the plan for the dressing regimen	Dressing change depends on the wound	Dressing routines	Adherence to protocols and commonly agreed praxis
“My issue was that according to some, you know, here we deal with frequently changed expats, okay. And everyone has almost a background, a medical background, and by that background by one way or another they influence the practice here.” 10^a^	Issue with frequently changing expats and their different backgrounds influencing the practice in the project.	Expats use different regimens	Mixed messages due to a high turnover of expats	Deviations from protocols due to uncertainty or due to deliberate decisions
“Well, I try my hard, my best to take, to, to, not to prescribe actually. AÄ: But sometimes it is difficult? P: Yeah, sometimes difficult, sometimes yeah” 11^a^	Difficult not to prescribe antibiotics when patient or relatives of patient insist	Insistent patient hard to resist	Deliberate antibiotic prescription deviations	Deviations from protocols due to uncertainty or due to deliberate decisions

^a^Participant ID

### Trustworthiness

To improve the trustworthiness, several aspects were taken into account. Parts of the interview material were coded by both AÄ and KKH and then compared to ensure codes were derived from the raw data and not affected by researcher bias. When discrepancies were encountered they were brought up for discussion within the research team. The differences in coding were discussed until the interpretation could be agreed upon. Member-check was implemented during the interview period, where AÄ continuously reviewed the material and went back to the participants if clarification was needed in order to completely understand the content. Peer-check was implemented primarily during the analysis and writing processes by continuous consultation with three experienced qualitative researchers.

## Results

One main theme emerged from the analysis: “Conflict between reality and adherence to protocols and commonly agreed praxis” (Table 3). The study findings are presented under each category/sub-category. The number following each quotation indicates participant ID. Contextual information for the quotations is presented by the author in squared brackets.

**Table 3:** A summary of categories and subcategories making up the theme “Conflict between reality and adherence to protocols and commonly agreed praxis”

Theme	Category	Sub-category
Conflict between reality and adherence to protocols and commonly agreed praxis	Adherence to protocols and commonly agreed praxis	Surgical interventions
		Dressing routines
		Antibiotic use
		Wound assessment and treatment of infection
	Inability to adhere to protocols	Lack of medication or equipment
		Lack of space
		Patient and caregiver behaviour
	Deviations from protocols due to uncertainty or due to deliberate decisions	Mixed messages due to a high turnover of expatriates
		Lack of consensus on hygiene routines
		Deliberate antibiotic prescription deviations
		Deliberate hygiene routine deviations

The main theme highlights the difference between theory and practice. The theme emerged from three categories: (i) Adherence to protocols and commonly agreed praxis, (ii) Inability to adhere to protocols, (iii) Deviations from protocols due to uncertainty or due to deliberate decisions.

### Adherence to protocols and commonly agreed praxis

This category was constructed from four sub-categories: (i) Surgical interventions, (ii) Dressing routines, (iii) Antibiotic use, (iv) Wound assessment and treatment of infection.

According to the participants, for war wound management there is both consensus and adherence when it comes to the principles of debridement and the evaluation of the general condition of the patient. These basic foundations were described as the cornerstones for the surgical management. Debridement was described as the primary intervention for both the prevention and treatment of local infection.

*“So, I think the best antibiotic and the best management [of war wounds] is a good debridement, and I can say it should be aggressive debridement.”* (3).

Participants stated that after the wound is surgically cleaned and fractures have been stabilised, most commonly with external fixation, the wound is generally left open for the first 3–5 days. The wound is dressed and the condition of both patient and wound is continuously re-evaluated with special regard for signs of infection, either local or systemic.

*“Yeah, the best things [in order to detect infection] is the close monitoring of vital signs of the patients… In the matter of temperature, if any raise or spikes of fever, if there is tachycardia, pain at the site of surgery, discharge, evaluation of the wounds from outside, the amount of discharge, if there is smelling. All these are signs of infection.”* (3).

Participants described the assessment of infection as being based on clinical examination of the wound, the general condition of the patient and tissue culture results. Tissue cultures are taken in the operating theatre when a patient shows signs of systemic infection.

*“Day one we start the patient as the protocol. If we see that after debriding the wounds, several debridements, the wound is still not clean… and has signs of inflammation [infection] and the patient is not stable, he’s having spikes of fever or… clinically not stable. Then we take cultures and we wait.”* (6).

Descriptions of the treatment of infections indicate that infections are managed through a combination of surgical and antibiotic treatment. Local infection is primarily treated by repeated debridement or rigorous local wound care, including increased frequency of dressing changes and irrigation.

*“… if we think that the wound is infected and it’s not systematic [systemic], so, we do the debridement and we keep trying debridement without antibiotic.”* (1).

Participants presented several general points on antibiotic treatment. The main aim of the physician was described as making the patient feel safe, taken care of and that it is important that the patients understand why an antibiotic prescription is or is not needed.

*“I’d probably take them back for another consultation for them to feel safe and to be able to give the information once again, or depending on what they want, but I would not prescribe antibiotics on indication that the patient just wants it.”* (7).

### Inability to adhere to protocols

Three main sub-categories were identified under this category: (i) Lack of medication or equipment, (ii) Lack of space, (iii) Patient and caregiver behaviour.

Participants felt forced to deviate from protocols at times of medication and equipment shortage in Jordan. The antibiotic protocol contains antibiotics that are sometimes not available. In these cases, the protocol cannot be adhered to. Insufficient availability of antibiotics recommended in the protocol was considered a major challenge in the treatment of infections and an indirect factor in the development of antibiotic resistance. One participant brought up the development of the protocols at headquarter level as a possible contributor to these problems. Interestingly, one participant described that “you know what is available between your hands” suggesting that the participants are aware and adapt in situations with limited resources. It indicates that participants learn what is available and modify their treatment strategies to make use of the resources that are available.

*“And also, there is some type of medication that it’s written in the protocol but it’s not here, such as the Rifampicin, which is used, it’s written in the protocol for using in some type of osteomyelitis for some bacterial […] osteomyelitis but it’s not here. So, there is some few gap…”* (2).

The participants identified the shortage of bed sheets as a cause of non-adherence to hygiene routines. Situations were described where the bed sheets could not be washed more than every 3–4 days because there were no clean sheets to replace the dirty ones. Participants stated that when clean bed sheets cannot be provided it is not possible to uphold the basics of good hygiene routines.

*“We have a gap of sheets. So, you can’t change your sheet but every three or four days.”* (6).

Lack of space was presented as a primary problem in adhering to hygiene routines due to over-crowded wards and close proximity of beds. Space also constitutes an issue when receiving patients in need of isolation.

*“We are very crowded. They [the beds] are very close together because we have limited space, limited rooms, and so there’s always a bed in addition so it’s really crowded.”* (4).

Some participants perceive the behaviour of patients and caregivers as a reason why hygiene routines cannot be followed. Patients tend to use everything in the wards and common spaces contemporaneously; they sit on each other’s beds or change beds. Patients can sometimes not manage to get to the facilities during the night and then excrement contaminates the bed sheets and the beds. The caretakers accompanying the patients crowd the wards further still.

*“… they [the patients] are using each other’s beds. And not only them, the caretakers are sleeping in their beds.”* (6).

The lack of space and the behaviour of patients and caregivers are viewed as possible contributors to healthcare-associated infections.

### Deviations from protocols due to uncertainty or due to deliberate decisions

This category is built from four sub-categories: (i) Mixed messages due to a high turnover of expatriates, (ii) Lack of consensus on hygiene routines, (iii) Deliberate antibiotic prescription deviations, (iv) Deliberate hygiene routine deviations.

Participants expressed uncertainty around the content of the protocols due to the various approaches used by different expatriates. These expatriates are often non-Jordanian physicians working in the MSF project. The reasons for these varying approaches were thought to be due to differences in the expatriates’ backgrounds and experiences from previous work. Various, often opposing instructions are sometimes given, primarily for dressing routines but also for antibiotic choices, which infection signs to look for and how to distinguish between contaminated and infected wounds. Some participants described how the protocols had been altered due to these recommendations and then rewritten again within a month. The participants stressed the importance of adhering to the protocols in order to minimise deviations, but concluded that this is not a simple task.

*“Different expats come and decide to start something new. Like using sugar, using chlorine, using wet-to-dry or just dry gauze. So, different point of views from the surgeons.”* (6).

Uncertainty was also described for hygiene routines. We identified a lack of consensus both in the interpretation and the adherence to the routines. For example, some participants described how staff wear long sleeves, rings and watches while on duty, but other participants contradicted this.

*“No, I don’t think so… No. AÄ: Are people wearing them [rings and watches]? P: Not much actually.”* (11).

*“We’re also in a cultural setting where people do use long sleeves for instance. Yeah, all of us [the staff], well not me but, yeah, people working here, independently of position…”* (7).

Antibiotic prescriptions and hygiene routines were regarded as challenges as the participants presented the difference between the protocols and reality. Participants exemplified situations in which they consciously deviated from either antibiotic prescription protocols or commonly agreed praxis. The reasons for these deviations differed: patients demand a certain treatment, the physician wishes to maintain a good doctor-patient relationship, or a sense that deviation from the protocol would provide the best treatment for the patient. Another aspect highlighted by the participants was that the physicians may at times want to impress the patient with an effective antibiotic and would therefore prescribe a broad-spectrum rather than a narrow-spectrum substance.

Some participants discussed the level of adherence to the hygiene protocols, both by themselves and by their colleagues. The participants stated that deviations from these protocols do occur. One reason being that the routines are not taken seriously by the staff, and another being that the staff are aware that some routines are in place primarily to protect themselves and not the patient.

*“We’re not taking it [the hygiene routines] too seriously and I know that the infection control is struggling with us. I think we need regular reminders.”* (6).

*“When you ask people about it [the hygiene routines] they seem to be pretty aware of that they do not do this for the patients’ sake but for their own protection…”* (7).

## Discussion

The main theme identified conveys that the challenges hospital-based physicians encounter in the management of war wounded civilians primarily relate to the adherence to protocols for wound care, antibiotic treatment, and hygiene. We identified parts of the protocols and praxis that participants seem to adhere to. These parts mainly consist of what could be considered commonly agreed principles of war wound surgery. All participants recognise thorough debridement, delayed primary closure and the necessity to assess the general condition of the patient as cornerstones of war wound management. These principles are largely based on long-standing experience from armed conflicts, such as the wars in Korea and Vietnam [5]. The views put forth by the participants suggest that this is a non-controversial foundation upon which they all rely.

We found that the main reasons for protocol deviation were either related to factors that the physicians cannot directly affect by themselves or situations where participants make a decision based on grounds other than medical, for example prioritising maintaining a good doctor-patient relationship over protocol adherence.

### Forced deviations from protocols

Forced deviations from the hygiene protocol were associated with high numbers of patients combined with limited space, leading to inadequate distance between beds and the inability to provide separate rooms for patients in need of isolation. Previous research indicates that the design of a hospital affects compliance with isolation protocols [18]. Our study participants described the lack of space as being associated with increased risk of spreading bacteria and a possible contributor to healthcare-associated infections, which seems to be consistent with existing research [19]. Even in a stable hospital setting, overcrowding is a challenge [20]. Levels of compliance to hygiene routines have been shown to decrease with increased crowding [21]. When managing a high number of war-injured patients, often with large open wounds, this becomes a major challenge. We found that patient influx and lack of space are perceived as factors beyond the control of the participants.

Absence of medications and equipment leads to deviations from the protocols. The World Health Organisation recognises this as a major concern worldwide, particularly in low- and middle-income countries [22]. The ICRC states that it is seldom the capabilities of the medical personnel that are lacking, but rather the technical and medical equipment that limits what can be done [5]. Our findings seem to support this perception.

Patient behaviour was described as another factor affecting adherence to protocols. This factor was found to be difficult for the staff to influence. We identified signs of frustration when participants described how both patients and caregivers ignored the rules and regulations of the hospital, making it impossible for the staff to adhere to the hygiene protocols. Similar results emerged from a previous study on clinicians’ perceived challenges in wound management, where frustration with patient non-compliance was one of the main findings [23].

### Conscious deviations from protocols

Conscious deviations from the protocols were described by the study participants. The participants did not stress the importance of following the details of the hygiene protocols, suggesting that the details were not found to be essential for patient management. It was also stated that the staff are of the opinion that much of the hygiene protocol was implemented not to protect the patients but to protect the staff and that the staff did not take the protocols seriously. There seem to be a discrepancy between the awareness of protocol details and what is practiced. Possible explanations for this could be a lack of time, a sense of powerlessness due to workload, or scepticism toward the importance of hygiene protocol adherence. Previous research has shown that compliance with hygiene routines diminishes with increased patient numbers [21] and that the compliance is highest after direct patient contact but lower after contact with the environment surrounding the patient [24].

The participants presented divided views in relation to the degree that they thought the protocols were being adhered to. Opposing views on whether staff wear long sleeves or not could be explained by participants not thinking of long sleeves under scrubs as actual “long sleeves”, or possibly that participants knew this practice to be wrong and therefore stating that it was not practiced. Cultural factors are also known to affect hand hygiene in different populations and religion may influence the attitude towards the use of alcohol-based hand cleansers, for example [25].

Deviations from antibiotic prescription protocols were found largely to be due to the wish of the physicians to maintain a good doctor-patient relationship. These results are consistent with findings in a study of factors influencing antibiotic prescription amongst physicians in rural India, where personal experience was a major influence on prescribing decisions and hospital guidelines were a minor influence [26].

### Uncertainty of protocol details

Another aspect complicating the adherence to protocols was the uncertainty of the actual protocols, particularly due to mixed instructions from foreign members of the medical team and the lack of consensus on the protocols amongst the staff. These mixed instructions were discussed and thought to be due to differences in background and training of the expatriates. Depending on the expatriates’ knowledge and experience, different methods of dressings and antibiotics were recommended. The inconsistency in these recommendations makes it difficult to maintain a stringent adherence to set protocols. The primary reason for these inconsistencies was stated to be that “reality rarely looks like the guidelines” and therefore a decision has to be made for each individual case. This individual evaluation could be seen as both a cause of deviation from the protocols and as a necessity for the correct treatment of the patient. Previous research has shown that obvious disparities between recommendations in the guidelines and the reality influence the perceived relevance, and subsequently the adherence [27]. It has also been demonstrated that when guidelines are perceived as simple and practical, the implementation is positively affected [28].

### Limitations

The qualitative methods used limits the generalizability. As the study was limited to one specific hospital, the findings may not be straightforwardly transferable to other contexts. However, a heterogeneous group of participants were selected to increase the transferability, as described above. The results may therefore be transferred to similar settings of war wound management, primarily in the same geographical region. Neither the researchers nor the interviewees have English as their first language. This could limit the participants’ ability to correctly express their views or result in misinterpretations. However, English is the language used in the project, why the risk of this affecting the results was considered to be small.

### Implications and future research

The knowledge gained by this study could provide valuable information for what aspects to consider when establishing future clinical projects and how to further improve the management of war wounds and the use of antibiotics in an unstable setting, such as a hospital in close proximity to armed conflict. The findings, for example difficulties in adherence to hygiene protocols and the problem of maintaining continuity associated with the frequent change of foreign physicians, could provide insights for the development of new protocols or guidelines for similar settings.

The findings and ideas generated by this study could generate hypotheses for further exploration in future qualitative or quantitative studies. A qualitative study using grounded theory with participants from the medical teams could be used to further explore the factors affecting adherence to protocols, and function as a first step towards resolving the discrepancy between guideline recommendations and actual practice.

## Conclusions

This study shows that adherence to established protocols around the management of war wounded civilians is difficult. In the population studied, there seems to be consensus and adherence to protocols pertaining to certain areas. These areas could be considered commonly agreed principles of war wound surgery, such as the use of wound debridement and the evaluation of the systemic condition of the patient prior to initiating antibiotic treatment. However, we have identified some challenges making it difficult or even impossible for the participants to follow the protocols. These challenges mainly consist of limitations imposed on the physicians, such as lack of space and materials, and the unfavourable behaviour of patients and caregivers. The participants also make conscious decisions to deviate from protocols, either to ensure that a good doctor-patient relationship is maintained or if they believe there are particular medical reasons why the protocols cannot be adhered to. Some of the identified protocol deviations could potentially lead to the development of healthcare-associated infections and antibiotic resistance. The knowledge gained by this study could provide insights when establishing future clinical projects and constructing protocols for wound management and antibiotic use in a similar setting.

## Appendix 1

### Introductory questions and probing areas

1. How did you manage your last patient with a war-associated soft tissue wound? Was this management similar or different compared to a typical case?
  *Probe*: Debridement, dressing type, dressing change frequency

2. What indication for antibiotic prescription was there for your last patient with a war-associated soft tissue wound? Was this management similar or different compared to a typical case?
  *Probe*: Common pathogens, differentiating between contaminating and infecting organisms, signs of infection, cultures, use of antibiotic prescription guidelines

3. How would you treat infection in war-associated soft tissue wounds?
  *Probe*: Antibiotic types, the use of broad-spectrum antibiotics

4. What is your view on antibiotic resistance?
  *Probe*: Possible causes, impact of antibiotic use, the use of broad-spectrum antibiotics

5. Have you ever prescribed antibiotics when there is no real indication, just in case?
  *Probe*: Influence by the patient or relatives

6. How do people obtain antibiotics?
  *Probe*: Without a prescription, Jordan, Syria, types of antibiotics, broad-spectrum antibiotics

7. What is your view on antibiotic resistance at Ar Ramtha hospital?
  *Probe*: Interviewees will be presented with antibiotic resistance data from Ar Ramtha hospital, possible causes, impact of antibiotic use, the use of broad-spectrum antibiotics

8. What is your view on hand hygiene?
  *Probe*: Gloves, disinfectants, in-between patients, dressings

9. What is your view on healthcare-associated infections?
  *Probe*: Possible causes, role of hand hygiene

10. How do you view the cooperation with the laboratory, regarding wound cultures?
  *Probe*: Sending cultures, patient information on referrals, receiving culture results, laboratory involved in treatment decisions, desired cooperation with the lab

11. Is there something you would like to add?

## Abbreviations

Expat: Expatriate, often a foreign member of the medical team
ICRC: International Committee of the Red Cross
MSF: Médecins Sans Frontières/Doctors Without Borders
